# Impact of Co_3_O_4_ nanoparticles on epoxy's mechanical and corrosion-resistance properties for carbon steel in seawater

**DOI:** 10.1038/s41598-024-53967-4

**Published:** 2024-02-12

**Authors:** M. A. Deyab, Omnia A. A. El-Shamy, Majed M. Alghamdi, Adel A. El-Zahhar

**Affiliations:** 1 Egyptian Petroleum Research Institute, Nasr City, Cairo 11727 Egypt; 2 Department of Chemistry, College of Science, King Khalid University, P.O. Box 9004, 61413 Abha, Saudi Arabia

**Keywords:** Co_3_O_4_-NPs, Epoxy, Corrosion, Coatings, Brine solution, Chemistry, Electrochemistry, Inorganic chemistry

## Abstract

Co_3_O_4_ nanoparticles (Co_3_O_4_-NPs) are synthesized using the facile solvothermal method. FT-IR and XRD spectroscopic analyses verify the creation of cobalt oxide nanoparticles with an average size of 13.20 nm. Furthermore, Zeta potential assessments were carried out to identify the electrical charge of the surface of the produced Co_3_O_4_-NPs, which was found to be -20.5 mV.  In addition, the average pore size of Co_3_O_4_-NPs is 19.8 nm, and their BET surface area is 92.4 m/g. The study also concerned the effect of Co_3_O_4_-NPs on epoxy's improvement of mechanical and corrosion protection for carbon steel in salt solution. By including Co_3_O_4_-NPs in an epoxy (EP) coating, corrosion is effectively prevented by non-permeable protective coatings that effectively reduce the transfer of corrosion ions and oxygen.

## Introduction

A highly regarded area of study is the protective methodology against steel corrosion in the surrounding sea. Researchers have extended carbon steel's durability by looking into various practical and affordable strategies, like corrosion protection using coatings^1,2^. To protect carbon steel from corrosion in the sea surrounding, it is crucial to create and improve coatings with preferable corrosion protection quality and reliable mechanical characteristics^3^.

One of the most often used transition metal oxides is cobalt oxide (Co_3_O_4_) nanoparticles because of their relatively high electrochromic efficiency (25 cm^2^ C^−1^), large surface area, strong conductivity, and enhanced electrochemical stability^4^. Cobalt oxide alone or in mixed form is used as protective thin film under different temperatures and pressures^5–7^.

A thermosetting polymer called epoxy resin (EP) is frequently utilized as a corrosion protection coating due to its excellent chemical compatibility in acid/base environments^8,9^. Because even though EP coating possesses fine chemical reliability, corrosion protection effectiveness, and moderate adhesion to carbon steel substrates, a few small cracks, microscopic pores, and pinholes will be produced in the coating with time^10,11^. These tiny pinholes and cavities make it simple for corrosive species to enter the carbon steel surface and lead to corrosion. The hydrated forms of aggressive ions can actually permeate coating flaws, resulting in the corrosion process^12–14^. This causes a rise in the concentration of H^+^ ions, which causes hydrogen evolution and, as a result, a decrease in pH.

Inorganic nanoparticles like carbon nanotubes (CNTs), Silicon dioxide, and TiO_2_ have frequently been incorporated into the EP protective layer as nanofiller to enhance protection against corrosion and lengthen the operational life of EP coating^15–17^. Khun et al.^18^ studied the effect of multiwall carbon nanotube “MWCNT” on corrosion resistance of epoxy/aluminum alloy composite. The authors concluded that with a higher MWCNT content, the epoxy composite coatings' adhesive strength increased. Due to improved solid lubricating and rolling effects of the MWCNTs and higher load-bearing capacity of the composite coatings, increased MWCNT content also decreased the friction coefficient and raised the wear resistance of the epoxy composite coatings.

Lately, Co_3_O_4_ nanoparticles (Co_3_O_4_-NPs) have received a lot of interest in different areas as biomedicine uses due to their eco-friendliness^3,19^. El-Shamy and Deyab^4^ are concerned with the importance of Co_3_O_4_ nanoparticles in corrosion performance in different studies^6,7,20^. Herein, Co_3_O_4_-NPs were prepared and characterized via XRD and FT-IR, then added as a nano-filler to EP coating to significantly improve the coating's barrier to the transfer of aggressive species. Electrochemical impedance spectroscopy and mechanical assessments were performed to examine the corrosion protection capability and adhesive pull of Co_3_O_4_-NPs@EP coating for carbon steel in a 3.5 wt.% brine solution.

## Experimental

### Materials

Cobalt nitrate hexahydrate (98%) was purchased from Aldrich (Chemical Corporation, USA). Cetyltrimonium bromide (CTAB, > 98% by TLC) was obtained from Merck (Germany). Sodium hydroxide and absolute ethanol (99%) are acquired from Fluka Chimica (Switzerland). Without further purification, all chemicals have been used.

The steel elements' percentage weights were C = 0.242, P = 0.043, Si = 0.301, S = 0.03, Mn = 0.482, and Fe = up to100.

### Synthesis of Co_3_O_4_-NPs

The solvothermal method^21^ was implemented for the preparation of cobalt oxide nanoparticles (Co_3_O_4_-NPs) by dissolving (0.024 mol, 0.678 g) of Co(NO_3_)_2_.6H_2_O in 12 ml of ethyl alcohol forming red color solution (A). In another 250 ml conical flask, prepare 0.09 mol of sodium hydroxide by dissolving appropriately in ethanol, forming a colorless solution (B). Then, solution (A) was added drop by drop to solution (B) during vigorous stirring using a magnetic stirrer. The previous mixture was allowed to complete for 20 min by forming blue color solution (C). The reaction was then completed by transferring mixture (C) after adding 0.6 g CTAB (prevent aggregation of the NPs) to 100 ml autoclave with Teflon linear and heated for five hours at 180 °C. Finally, black powder of Co_3_O_4_ nanoparticles was obtained after washing several times using ethanol and drying in the oven.

### Preparation of Co_3_O_4_-NPs@EP coating

The Co_3_O_4_-NPs@EP coating was created by combining EP resin from Ciba, poly-amidoamine (hardener) from Arkema, and 2.5 wt.% Co_3_O_4_-NPs. When a high concentration of Co_3_O_4_-NPs is added (more than 2.5 wt.% Co_3_O_4_-NPs), agglomerates form, causing the dispersion to be deemed insufficient to be used any further quantity.

The EP resin to hardener weight ratio became 2:1. All of the components were blended for 3 h with a speed mixer (1300 rpm)^22,23^. High-purity N_2_ flow was bubbled into the mixture while stirring.

**Figure 1:**
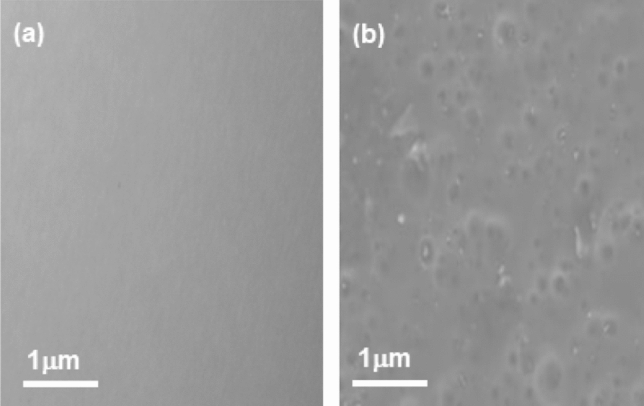
SEM images (**a**) neat EP resin, (**b**) Co_3_O-NPs@EP coating.

The uniform dispersion of Co_3_O_4_-NPs in EP resin was checked using a scanning electron microscope (ZEISS scan electron microscopy, SEM). Figure 1a illustrates that the surface of the neat EP resin is smooth and free of impurities. In contrast, spherical particles are visible on the Co_3_O-NPs@EP surface (Fig. 1b). In EP resin, the Co_3_O_4_-NPs particles were dispersed uniformly.

Before coating, the metal sheets were degreased with acetone and ultrasonic cleaning with 95% ethanol, then with de-ionized water and drying.

The clean substrate was covered with Co_3_O_4_-NPs@EP coatings using a film applicator^24^. The dry film thickness of EP resin and Co_3_O_4_-NPs@EP coating was determined using a coating micrometer of 45.2 ± 3 and 63 ± 4 μm, respectively.

### Corrosion and mechanical tests

As the working electrode, a carbon steel panel was employed to evaluate the resin's resistance to corrosion in 3.5% NaCl solution.

The EIS was applied using a Gamry-Interface-5000E potentiostat/galvanostat to investigate the coating system's effectiveness. For EIS experimental tests, an electrochemical cell defining a set was used. The platinum (counter electrode), coated carbon steel (working electrode), and saturated calomel (reference electrode) electrodes were used to build the cell.

The exposed surface of the coating is one side with a dimension of 30 mm × 10 mm during the EIS measurement. Impedance spectra were recorded at open circuit potential (6 h) with frequencies ranging from 1 Hz to 30 kHz and potential amplitudes equal to 10 mV. After seven days of immersion, the test was carried out. The nano-indentation method was used to evaluate mechanical characteristics (Micro Materials instruments). Using Micro Materials instruments, the nano-indentation test was carried out on the nanocomposite sample in three stages: loading, holding, and unloading^25^. The international standard ISO 14577 governs these experiments. Impact resistance and scratch-hardness tests were performed by ASTM specifications (ASTM-D2794, ASTM-D7027)^26^. The experiments were done in triplicate, and the outcomes were very reproducible.

The salt spray experiment (3.5 wt.% solution) was performed in a corrosion tester cabinet at 323 K in accordance with ASTM B117. After 168 h, the samples surfaces and degree of rusting were inspected visually and evaluated.

### Methods of characterization

FT-IR spectroscopic measurement was obtained using a Perkin Elmer-Spectrum spectrophotometer on the prepared Co_3_O_4_-NPs (KBr pellet technique). Spectral data were gathered from 400 to 4000 cm^−1^. Functional reference spectra were used to determine groups. The produced nao-metal oxide' crystallinity was proven using an X-ray diffractometer (XRD). The applied wavelength (λ) was 1.5418 Å using Cu Kα radiation (PANalytical XPERT PRO MPD, Netherlands). At room temperature, the diffraction pattern is recorded in the angular range (2θ) of 10–80 with a step size of 0.02. In addition, zeta potential measurements (Malvern Zetasizer ZS-HT, UK) were used to identify the anti-aggregation resistance of the synthesized metal oxide nanoparticles. The measures depend on the electrophoresis where the value of zeta potential is related to the electrophoresis (Henry equation)^27^.

The N_2_ gas adsorption/desorption performance and specific surface area of the resulting material were studied using Brunauer–Emmett–Teller (BET) analysis with a Quantachrome NOVA Station A.

## Results and discussion

### Characterization of Co_3_O_4_-NPs

**Figure 2:**
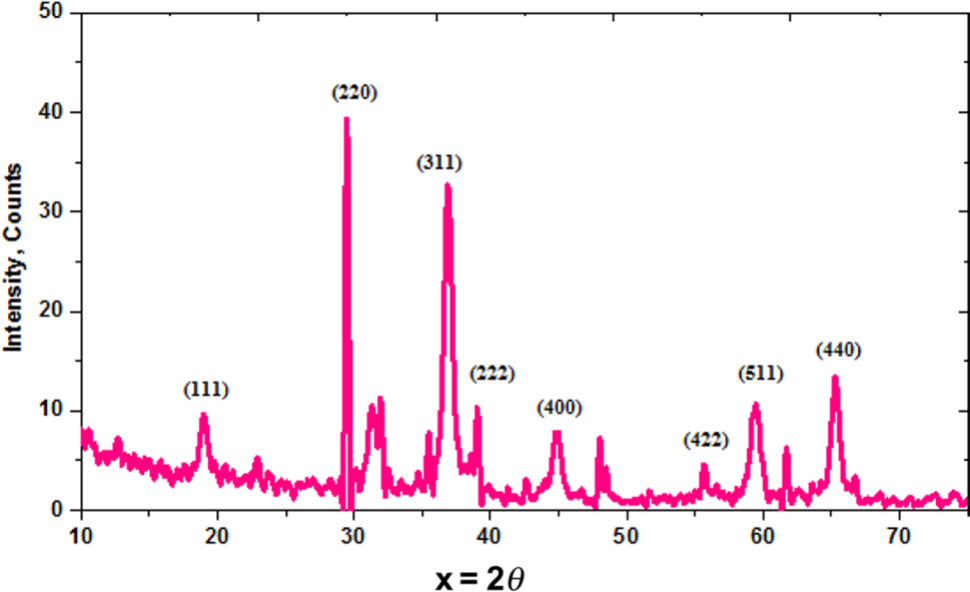
XRD spectrum of Co_3_O_4_-NPs.

Figure 2 declares the Co_3_O_4_-NPs characteristic XRD pattern, which confirms their crystallinity and crystal structure^28,29^. The observed diffraction peaks supported by the planes (111), (220), (311), (222), (400), (422), (511), and (440), which are all associated with the sharp diffraction peaks found at 2θ values of 18.99°, 29.56°, 36.95°, 39.04°, 44.79°, 55.64°, 61.61°, and 65.21°, which are in good accord with the JCPDS card No. 43-1003^30^.

Debye-Scherer’s equation determined the average particle size (D) of Co_3_O_4_-NPs^31^.

1 $$D \, = \, \frac{0.9\lambda }{{\beta \cos \theta }}$$

where, λ is the wavelength of the X-ray radiation source (0.15405 nm), *θ* is the half diffraction angle (also known as the Bragg angle), and *β* is the full width at half-maximum value (FWHM) in radians of the XRD diffraction lines. The previous equation declares that the peak width, as measured perpendicular to the reflecting planes, is inversely related to crystal thickness^32^. The mean diameter of the synthesized Co_3_O_4_-NPs is calculated and found to be 13.20 nm depending on (311).

**Figure 3:**
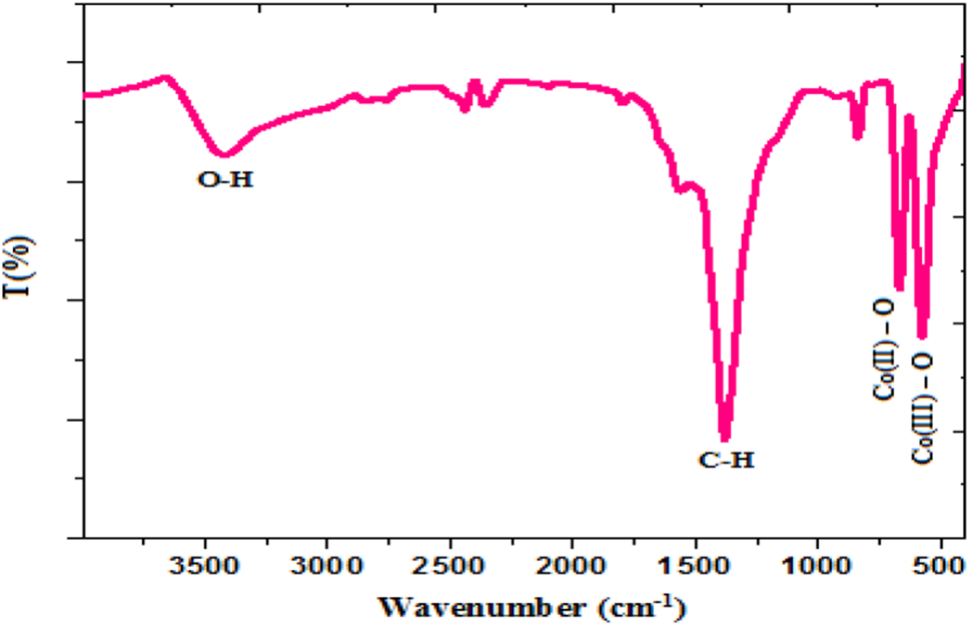
FT-IR pattern of the synthesized Co_3_O_4_-NPs.

The recorded FT-IR spectrum (Fig. 3) is used to obtain structural data from the functional groups of the produced Co_3_O_4_-NPs. Strong metal oxide bands at 666.63 cm^−1^ and 574.03 cm^−1^ are related to Co(III) in an octahedral and Co(II) in a tetrahedral site^33,34^. In addition, the peak present at 1390 cm^−1^ regards to C-H bending of CTAB that capped the formed nanoparticles^35^.

**Figure 4:**
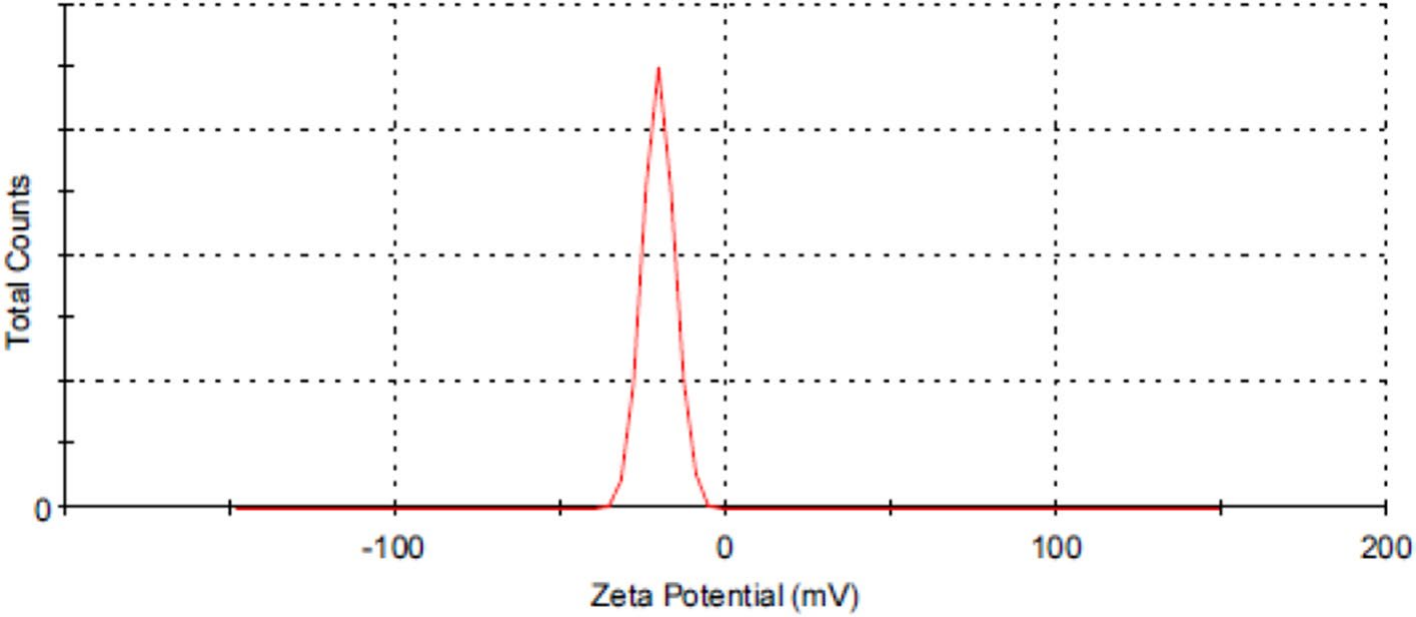
Zeta potential distribution of Co_3_O_4_-NPs.

Zeta potential is a physical property defined as the electrical potential between the investigated material and the surrounding liquid^36^. Figure 4 shows the zeta potential distribution with a mean value of −20.5 mV. Zeta potential is a good tool that can determine the particles' electrical surface charge^37^. When an electric field is applied over the scattered Co_3_O_4_-NPs, the nanoparticles will move toward the oppositely charged electrode at a rate proportional to the zeta potential. High zeta potential for a dispersion system, whether positive or negative, suggests that the particles are resistant to aggregation and indicates an apparently stable system^38^. The surface of synthesized Co_3_O_4_-NPs is the negative value of zeta potential which agrees with the literature^39,40^.

Co_3_O_4_-NPs have a BET surface area of 92.4 m^2^/g and an average pore size of 19.8 nm.

### Corrosion protection features of Co_3_O_4_-NPs@EP coating

**Figure 5:**
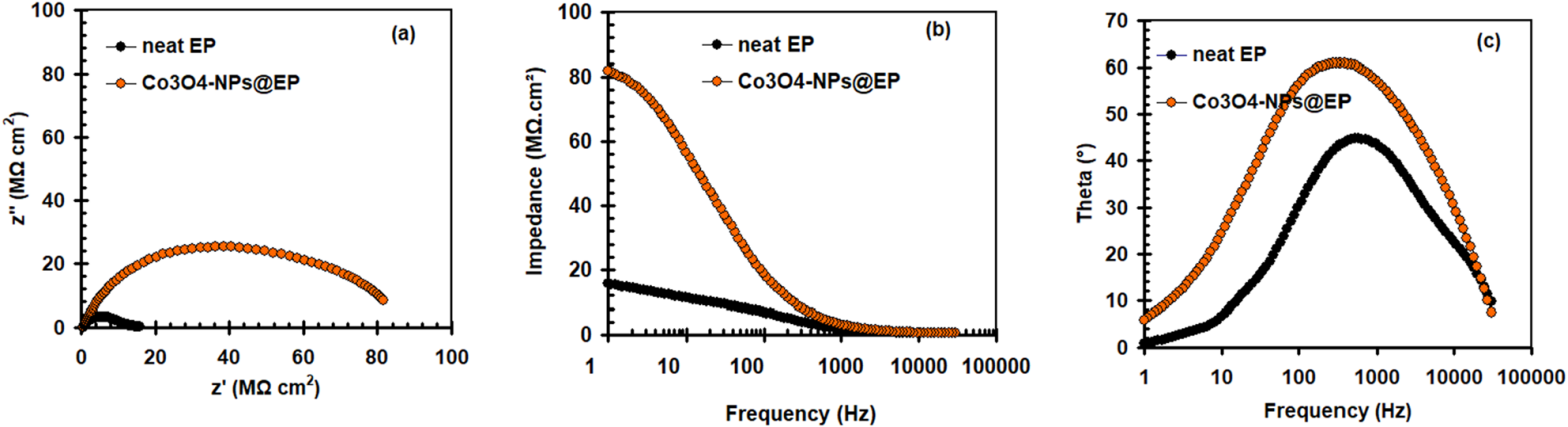
EIS spectra (**a**) Nyquist, (**b**) Bode-module, (**c**) Bode-phase angle plots for carbon steel coated with neat EP and Co_3_O_4_-NPs@EP coating in 3.5% NaCl liquid at 298 K after 7 days of immersion.

**Figure 6:**
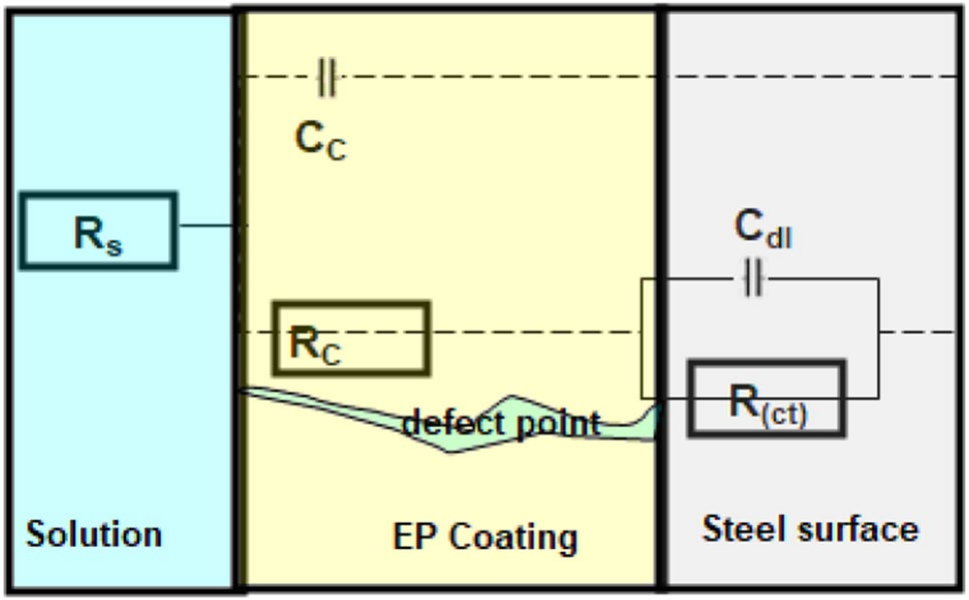
Equivalent circuit for fitting of the impedance data.

This section used EIS plots to evaluate the effectiveness of EP-coated carbon steel in both the incorporation and absence of Co_3_O_4_-NPs in 3.5% NaCl solution (see Fig. 5). As depicted in Fig. 5, two capacitive loop circuits make up the Nyquist, Bode-module, and phase angle plots for both coatings (Fig. 5a–c). The two capacitive circuits at high and low frequencies are induced by the capacitance and resistivity of the EP covering and the steel/electrolyte interface, respectively^41,42^. Figure 6 depicts an equivalent electric circuit that utilizes two-time constants for both coatings. Electrolyte resistance (*R*_s_), charge-transfer resistance (*R*_ct_), coating resistance (*R*_c_), coating capacitance (*C*_c_), and double-layer capacitance (*C*_dl_) are all present in this structure^43^. The coating capacitive circuits' radius was extended by adding 2.5 wt.% Co_3_O_4_-NPs (Fig. 5). The *R*_c_ and *C*_*c*_ qualities for neat EP coating are 15.3 MΩ cm^2^ and 1.6 × 10^–8^ F cm^−2^, respectively. In addition to an increase in *R*_c_ to 84.4 MΩ cm^2^ and a decrease in *C*_c_ value to 0.78 × 10^–9^ F cm^−2^, in the Co_3_O_4_-NPs@EP coating. Co_3_O_4_-NPs coating seemed to have a greater phase angle than a neat EP coating, denoting that it could be quite resistant (see Fig. 5c). In the case of neat EP coating, it is nearly impossible to prevent significant moisture transport through coating layers to control the corrosion process^10^. The electrochemical processes that take place at the defect points in the coating layer can be outlined using the equations below^44,45^:

2 $${\text{Fe}}_{{({\text{s}})}} \to {\text{ Fe}}^{{{2} + }} + {\text{ 2e}} {\text{ at anodic sites}}$$

3 $${\text{O}}_{{2}} + {\text{ 2H}}_{{2}} {\text{O }} + {\text{ 4e}} \, \to {\text{ 4OH}}^{ - } {\text{at cathodic sites}}$$

When Co_3_O_4_-NPs are added to an EP coating, impermeable protective coatings are created that effectively block the transfer of corrosion ions and oxygen, preventing corrosion from occurring.

Co_3_O_4_-NPs can act as barriers within the epoxy matrix, hindering the movement of corrosive species such as water, oxygen, and ions. This physical barrier reduces the contact between the metal substrate and the corrosive environment, thereby slowing down the corrosion process^46^.

**Figure 7:**
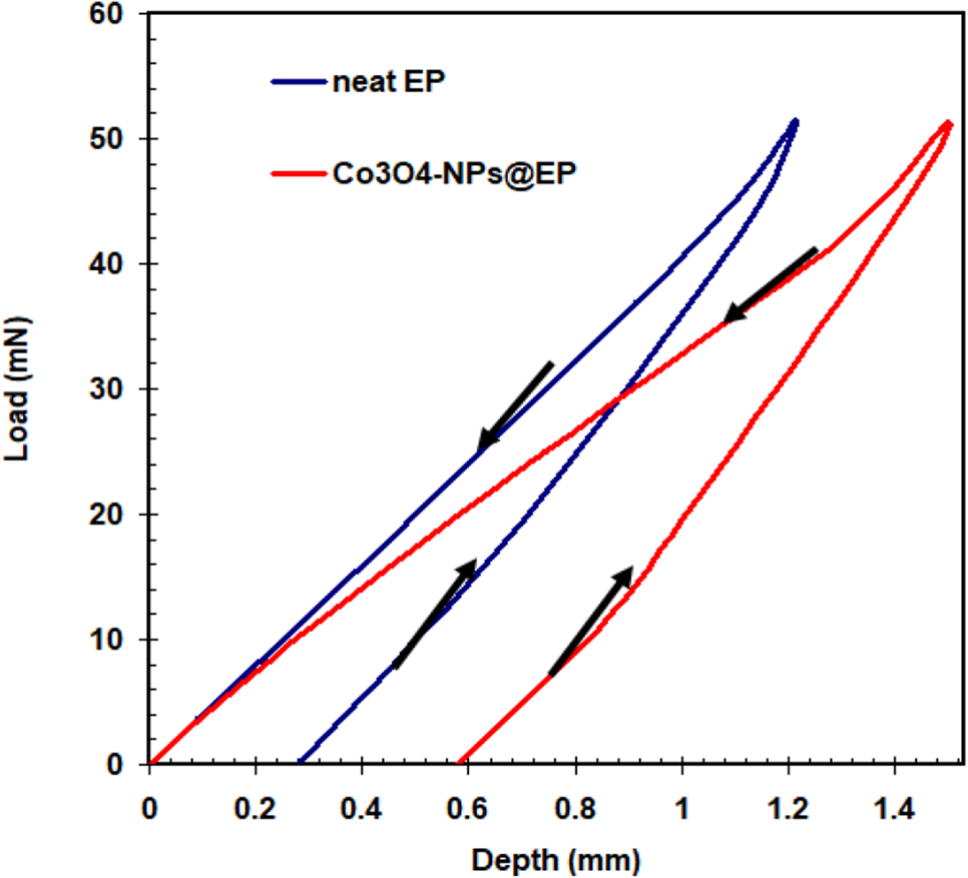
Loading–unloading charts for carbon steel surfaces coated with neat EP and Co_3_O_4_-NPs@EP.

Using the nano-indentation method, the mechanical characteristics of carbon steel with EP coating in both the absence and addition of Co_3_O_4_-NPs were examined. Loading–unloading charts for carbon steel surfaces coated with neat EP and Co_3_O_4_-NPs@EP are seen in the Fig. 7.

Evidently, the EP coating adapted with Co_3_O_4_-NPs provided markedly improved EP coating hardness (from 126 to 445 mN/m^2^). This trend could be attributed to the Co_3_O_4_-NPs' propensity to fill in cracks and open spaces in the EP coating, which lowers the overall free volume and raises the cross-linking density of the dried epoxy^47,48^. The physicomechanical features of carbon steel surfaces coated with neat EP and Co_3_O_4_-NPs@EP are described in Table 1.

**Table 1:** Physicomechanical features of carbon steel surfaces coated with neat EP and Co_3_O_4_-NPs@EP.

Impact-resistance (kJ cm^−2^)	Scratch-hardness (GPa)	Type of coating
42.2	5.6	Neat EP
78.5	8.9	Co_3_O_4_-NPs@EP

It's worth noting that incorporating Co_3_O_4_-NPs into the neat EP positively affects the EP coating's scratch resistance. The scratch hardness of Co_3_O_4_-NPs@EP was the highest. The growth in scratch hardness could be associated with a decline in indentation caused by rising physical interaction between the EP resin and the Co_3_O_4_-NPs. Co_3_O_4_-NPs appear to be included in the EP polymer backbone to restrict chain mobility, leading to high-impact resistance (see Table 1). The dispersion of Co_3_O_4_-NPs within EP resin and the powerful interactions of Co_3_O_4_-NPs and EP resin with the epoxy polymer are the main considerations for improving the mechanical features of Co_3_O_4_-NPs@EP coating^49,50^.

**Figure 8:**
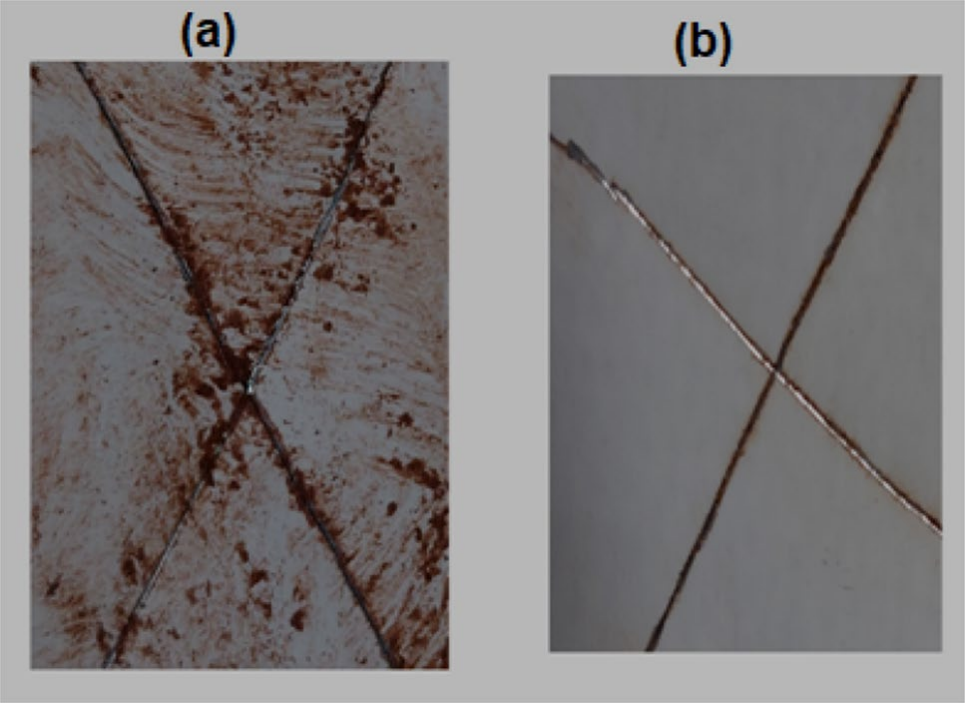
Photographs of cyclic salt-spray test in the case of (**a**) EP resin and (**b**) Co_3_O_4_-NPs@EP.

The salt spray experiment was carried out for 168 h to assess the impact of Co_3_O_4_-NPs introduction on the corrosion inhibition behavior of the EP resin coating. The various observable corrosions that formed across the scratch, as seen in Fig. 8a, indicate that neat EP seems to have poor corrosion inhibition features. The addition of Co_3_O_4_-NPs to the EP coating significantly minimized the degree of corrosion throughout the scratch of the coating (Fig. 8b). As a result, adding Co_3_O_4_-NPs will improve the compactness and the corrosion inhibition of EP coating.

## Conclusion

Co_3_O_4_-NPs were prepared by solvothermal method and then analyzed by FT-IR and XRD spectroscopic measurements. In a brine solution, the effect of Co_3_O_4_-NPs on the improvement of epoxy's corrosion protection and mechanical performance for carbon steel was investigated. For a neat EP coating, the *R*_c_ and *C*_*c*_ qualities are 15.3 MΩ cm^2^ and 1.6 × 10^–8^ F cm^−2^, respectively. Two capacitive circuits can be seen in the Co_3_O_4_-NPs@EP coating, with *R*_c_ increasing to 84.4 MΩ cm^2^ and *C*_c_ decreasing to 0.78 × 10^–9^ F cm^−2^. It was clear that the EP coating that had been modified with Co_3_O_4_-NPs increased the hardness of the EP coating (from 0.126 to 0.445 GPa). Incorporating Co_3_O_4_-NPs into an epoxy (EP) coating produces non-permeable protective layer that effectively inhibits the transmission of corrosion ions and oxygen, stopping corrosion.

## Data Availability

The datasets used and/or analysed during the current study available from the corresponding author on reasonable request.
